# Functional characteristics of a novel odorant binding protein in the legume pod borer, *Maruca vitrata*

**DOI:** 10.1038/s41598-021-93382-7

**Published:** 2021-07-07

**Authors:** Hui Ai, Yuying Liu, Guangyan Long, Yuan Yuan, Shaopei Huang, Yan Chen

**Affiliations:** 1 Hubei Key Laboratory of Genetic Regulation and Integrative Biology, School of Life Sciences, Central China Normal University, Wuhan, 430079 China; 2 Wuhan Donghu University, Wuhan, 430212 China

**Keywords:** Biochemistry, Biological techniques, Biotechnology, Zoology

## Abstract

Insect olfaction system plays a key role in the foraging food, pollination, mating, oviposition, reproduction and other insect physiological behavior. Odorant binding protein are widely found in the various olfactory sensilla of different insect antennae and involved in chemical signals discrimination from natural environment. In this study, a novel *OBP* gene, *MvitOBP3* is identified from the legume pod borer, *Maruca vitrata*, which it mainly harms important legume vegetables including cowpea, soybean and lablab bean. Real-time PCR results demonstrated that *MvitOBP3* gene was abundantly expressed in the antennal tissue of *M. vitrata*, while low levels were distributed in the head, thorax, abdomen, leg and wing of adult moths. The recombinant OBP3 protein was purified using the prokaryotic expression and affinity chromatography system. Fluorescence competitive binding experiments indicated that that MvitOBP3 protein exhibited greater binding affinities with host-plant flower volatiles including Butanoic acid butyl ester, Limonene, 1H-indol-4-ol and 2-methyl-3-phenylpropanal, highlighting they may have attractant activities for the oviposition of female moths on the legume vegetables. Moreover, protein homology modeling and molecular docking analysis revealed that there are six amino acid sites of MvitOBP3 involved in the binding of the host-plant volatiles. These findings will further promote to understand the key role of odorant binding protein during host perception and oviposition of *M. vitrata* moths, which improve the efficiency of semiochemical-based prevention and monitoring for this pest in the legume vegetables field.

## Introduction

Through efficient chemical communication, insects have an enormous impact in agriculture and forestry, which their olfaction are primary essential sensory input that regulates mating and reproduction. Insects can distinguish different odorant molecules from partner, host plants and prey through antennae to seek food, mating partners and oviposition sites^1–3^. External chemicals including host-plant volatiles and sex pheromones enter into the chemosensilla and then are captured by odorant binding proteins (OBPs) in the antennae^4,5^. OBPs are water-soluble olfactory protein molecules and consist of general odorant binding proteins (GOBPs) and pheromone binding proteins (PBPs)^6–8^. Generally, OBPs can carry lipophilic volatiles of host and prey to the olfactory receptor (Ors) cells through hemolymph circulation of antennae and function in the first step of insect odor reception^9^. PBPs are mainly involved in the chemical communication of sex pheromones between male and female pests and beneficial insects, which are produced in the pheromone glands (PG) located on the abdomen terminals^10^.

Currently, a large number of OBPs have been identified from the Lepidoptera, Hymenoptera, Coleoptera, etc. Zhang et al. reported that *Locusta migratoria* OBP4 protein was strongly bound with the male-specific 2-heptanone that differ between adult male and female migratory locusts, which providing new understanding of locust courtship and development of new control technologies^11^. AcerOBP2 was identified from Eastern honey bee (*Apis cerana*) and associated with olfactory recognition of floral volatiles and bee pheromones, which plays dual roles in pollination and reproduction of honeybee population^12^. A ligand binding assay revealed that recombinant *Agrotis ipsilon* GOBP1 and GOBP2 proteins highly bound not only to sex pheromone components but also to some volatile compounds from host-plant, which could further elucidated their olfactory molecular mechanism to distinguish different odorant molecules^13^. Besides, Li et al. reported that *Adelphocoris fasciaticollis OBP11* gene distributed below the base of antennal chemosensilla and exhibited stronger binding characteristics to non-volatile secondary metabolite compounds than the volatile odors, highlighting that it may play a vital role in the gustatory perception^14^. These findings together with other insect OBPs in the literature provided useful information for exploring chemosensory mechanism and integrated pest management strategies in the field.

The legume pod borer, *Maruca vitrata* (Lepidoptera, Crambidae) is one of the most serious pest of grain legumes in the tropics and subtropics, including Asia, Africa, Australia, America and Oceania^15^. The female moths like to visit the flowers and buds of legumes and lay eggs on them, while the *M. vitrata* larvae easily feed on the beans and stalks after hatch^16^. Several legume crops including cowpea, soybean, lablab bean, adzuki bean and black gram are threatened by this boring pest, which they all cause different levels of production hazards and pesticide residues^17–21^. Presently, conventional chemical pesticides are the main control strategy for the borer, but the pesticide residues in the leguminous vegetables may threat the health of the consumer and also does not comply with current general environmental policies. Therefore, it is urgent to explore new prevention strategies, such as sex pheromones and host-plant volatiles have been developed as alternative means of controlling pests, involved in mating disruption and field monitoring.

Our earlier findings indicated that the *M. vitrata* female moths preferred to lay eggs on flower buds/flowers of the host plants by the seventeen host-plant volatiles using the gas chromatography-mass spectrometry (GC–MS) and coupled GC-electroantennographic detection^16^. Moreover, we also found MvitPBPs proteins play an important role in binding four sex pheromone ligands of *M. vitrata*^22^. And MvitGOBP1-2 proteins could also greatly bind partial odorant molecules from seventeen kinds of host-plant flower volatiles^7^. In this study, a novel *OBP* gene was identified from the antennal libraries of *M. vitrata* and Real-time PCR method was used to explore its expression pattern in different tissues. Prokaryotic expression, affinity chromatography purification, fluorescence competitive binding assay and molecular docking will also be used to study the physiological functions of this *OBP* gene. These findings will further promote to understand the key role of odorant binding protein during host perception and oviposition of *M. vitrata* moths, which improve the efficiency of semiochemical-based prevention and monitoring for this pest in the legume vegetables field.

## Materials and methods

### Experimental materials

The *M. vitrata* larvae and adult moths were reared in incubators set at 26 ± 1 °C, 60% ± 10% relative humidity and L 14 h: D 10 photoperiod in our laboratory. All synthetic host-plant volatiles and sex pheromones in this study were purchased from Sigma Chemical Co. and Tokyo Chemical Industry Co.

### Recombinant expression of *M. vitrata OBP3* gene

The first strand of cDNA was synthesized using the FastQuant cDNA first strand synthesis kit. The *M. vitrata OBP3* gene was amplified by the primers according to the gene sequence (Genebank number: MK549108.1) and purified PCR products were connected with the pEASY-T1 vector under the reaction condition of 4 °C overnight. Signal peptide sequence of protein was predicted by SignalP 5.0. The PCR products of OBP containing restriction sites and the expression plasmid PET32a ( +) were digested with BamHI and XhoI. After enzyme digestion, the target gene and the expression vector were connected with T4 ligase at 4 °C overnight, and then the recombinant vector was transformed into *Escherichia coli* DH5a competent cells, coated with plate, and screened single colony sequencing verification. The successfully connected recombinant plasmid was transformed into *Escherichia coli* BL21 (DE3) competent cells. Appropriate temperature and IPTG concentration were selected to express a large number of recombinant proteins (37 °C, 220 rpm), and the 1.2 L recombinant strain was cultured with 1:100 inoculation proportion. After induction, the bacteria were centrifuged at 4 °C at 10,000 rpm for 20 min to collect the bacteria. Every 600 mL of bacteria collected was re-suspended with 30 mL PBS buffer and broken in a cryogenic and high-pressure crushing machine. Since the target protein was expressed in the supernatant, the supernatant was centrifuged at 10,000 rpm at 4 °C for 20 min after crushing, and then the recombinant protein was purified by affinity chromatography column (Huijin pro Ni-6FF(IDA)). The purified protein fractions were analyzed by SDS-PAGE gel electrophoresis.

### Fluorescence binding assays

The fluorescence measurement takes advantage of the fluorescent properties of the dye, 1-NPN (N-Phenyl-1-naphthylamine, CAS 90-30-2) and is performed by Hitachi F-4500. The wavelength of excitation light was 337 nm, the range of scanning emission wavelength was 350–500 nm, and the slit width was 5 nm. 1-NPN exhibits a detectably altered emission spectrum when interacting with the ligand-binding pocket of OBP3. Add 2 mL of the protein with a final concentration of 2 μmol/L to a 1 cm, four-sided quartz cup, then add 1-NPN (1 mM/L) to the final concentration is 2–24 μmol/L. And recorded the maximum fluorescence value and determine the protein dissociation constant K_1-NPN_.

### Tissue expression pattern of *MvitOBP3* gene

Quantitative real-time PCR (qRT-PCR) method was used to measure tissue-specific mRNA expression pattern of *MvitOBP3* genes on the Bio-Rad CFX 96 real-time PCR system with SYBR Green I fluorescent dye. Transcript abundance was determined for multiple tissues (antenna, heads, thoraxes, abdomens, legs, wings) from male and female moths. Each sample was carried out in three biological replicates and three technical biological replicates in real-time PCR reaction. qRT-PCR primers (OBP3YF, OBP3YR, actinYF and actinYR) were used to determine the relative abundance of *OBP3* mRNA in different tissues of *M. vitrata* (actin gene as the control) (Table 1). The quantitative real-time PCR was conducted in 20 μL reactions and analyzed by 2^−△Ct^ method.

**Table 1:** Primers used for prokaryotic expression and quantitative RT-PCR of *OBP3* gene.

Primer name	Sequence (5′–3′)
OBP3-BamHIF	CGCGGATCCATGGCCACCGCTCCCTA
OBP3-HindIIIR	CCGCTCGAGTTACAGGAAGATGGCGTGC
OBP3-YF	ACGGAGAATGATGCCCTGA
OBP3-YR	CGGCGAGTTGCCTTTGT
ActinF	AGCACGGTATCATCACCAACT
ActinR	GGTCTCAAACATGATCTGGGT

### Molecular docking of MvitOBP3 protein with host-plant volatiles

Three-dimensional model of MvitOBP3 was constructed using the online program SWISS MODEL (http://swissmodel.expasy.org/) and displayed by PyMOL Viewer (http://www.pymol.org/). The structure of the OBP56 of blowfly *Phormia regina* (5dic.1.A) was used as a template according to RCSB Protein Data Bank (PDB: http://www.rcsb.org/pdb/home/home.do). The protein alignment was carried out with DNAMAN software and ESPript (http://espript.ibcp.fr/ESPript/ESPript/). Based on the generated homology model and corresponding binding ligand, docking program AutoDock Vina was used to find the potential binding mode between MvitOBP3 protein and the effective host volatiles. Three-dimensional structure of the ligand was obtained from ZINC (http://zinc.docking.org/).

## Results and discussion

### Sequence analysis and phylogenetic tree construction of MvitOBP3

**Figure 1:**
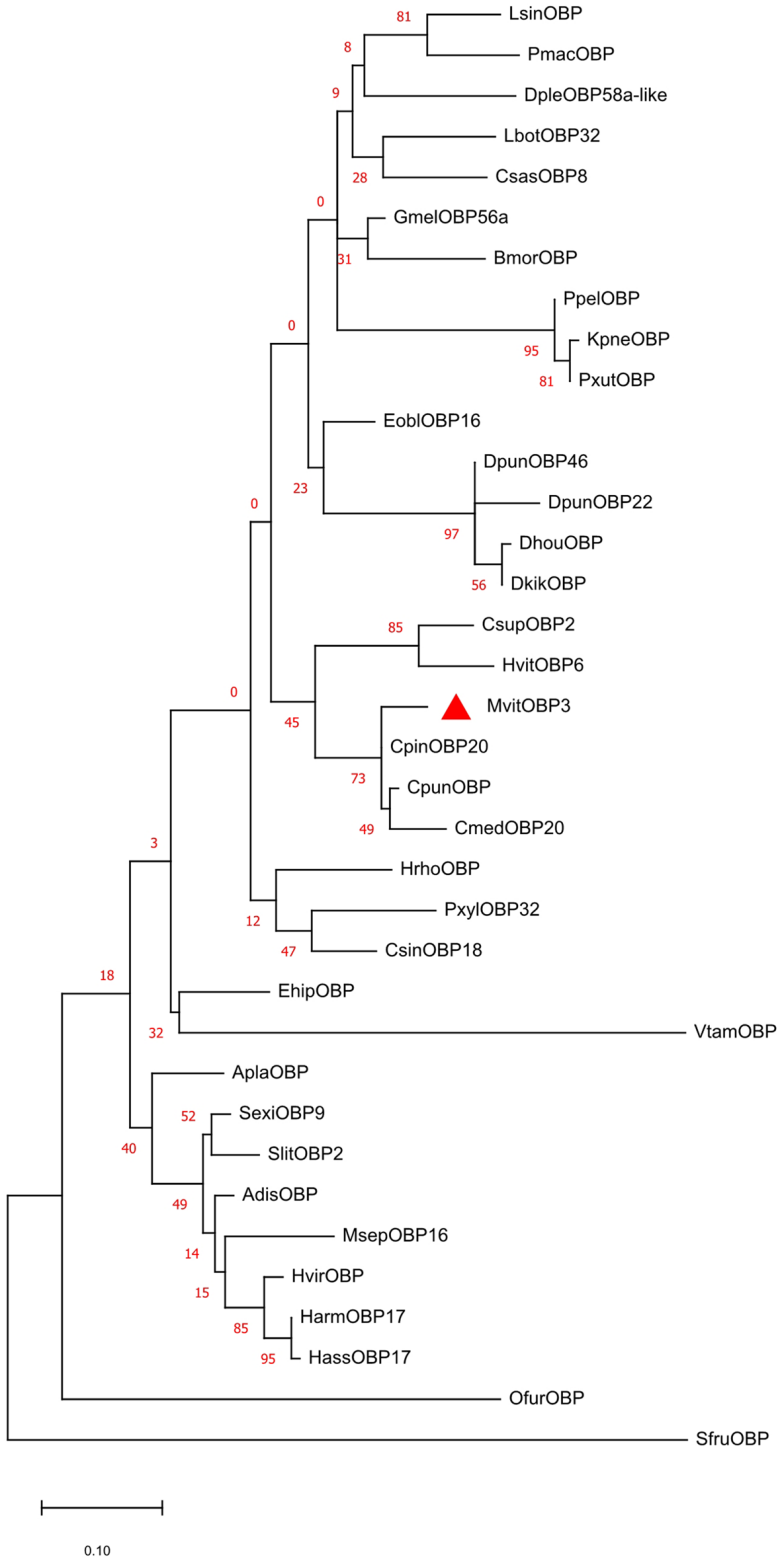
Evolutionary analysis of MvitOBP3 protein with other Lepidopteran insect OBPs. Genbank accession numbers: LsinOBP (VVC93553.1), PmacOBP (CAF4841106.1), BmorOBP (NP001040212.1), CsasOBP8 (AYD42183.1), LbotOBP32 (AXF48729.1), PxutOBP (NP001299556.1), DpleOBP58a-like(XP 032522559.1), GmelOBP56a (XP026763748.1), PpelOBP (WP143520259.1), KpneOBP (WP142379627.1), EoblOBP16 (ALS03864.1), DpunOBP46 (ARO70205.1), DpunOBP22 (ARO70181.1), DhouOBP (AII00983.1), DkikOBP (AII01006.1), CsupOBP2 (AGK24578.1), HvitOBP6 (AZB49387.1), CpinOBP20 (QEE82719.1), CpunOBP(AMY16434.1), CmedOBP20 (ALT31650.1), HrhoOBP (QGN01742.1), PxylOBP32 (ANG08535.1), CsinOBP18 (QGN03648.1), EhipOBP (AOG12864.1), VtamOBP (XP026497454.1), AplaOBP (CAB3233796.1), SexiOBP9 (AGH70105.1), SlitOBP2 (XP 022815401.1), AdisOBP (QCF41928.1), MsepOBP16 (AWT22226.1), HvirOBP (PCG75714.1),HarmOBP17 (AFI57166.1), HassOBP17 (AGC92792.1), OfurOBP (XP028157948.1), SfruOBP (KAF9813770.1).

**Figure 2:**
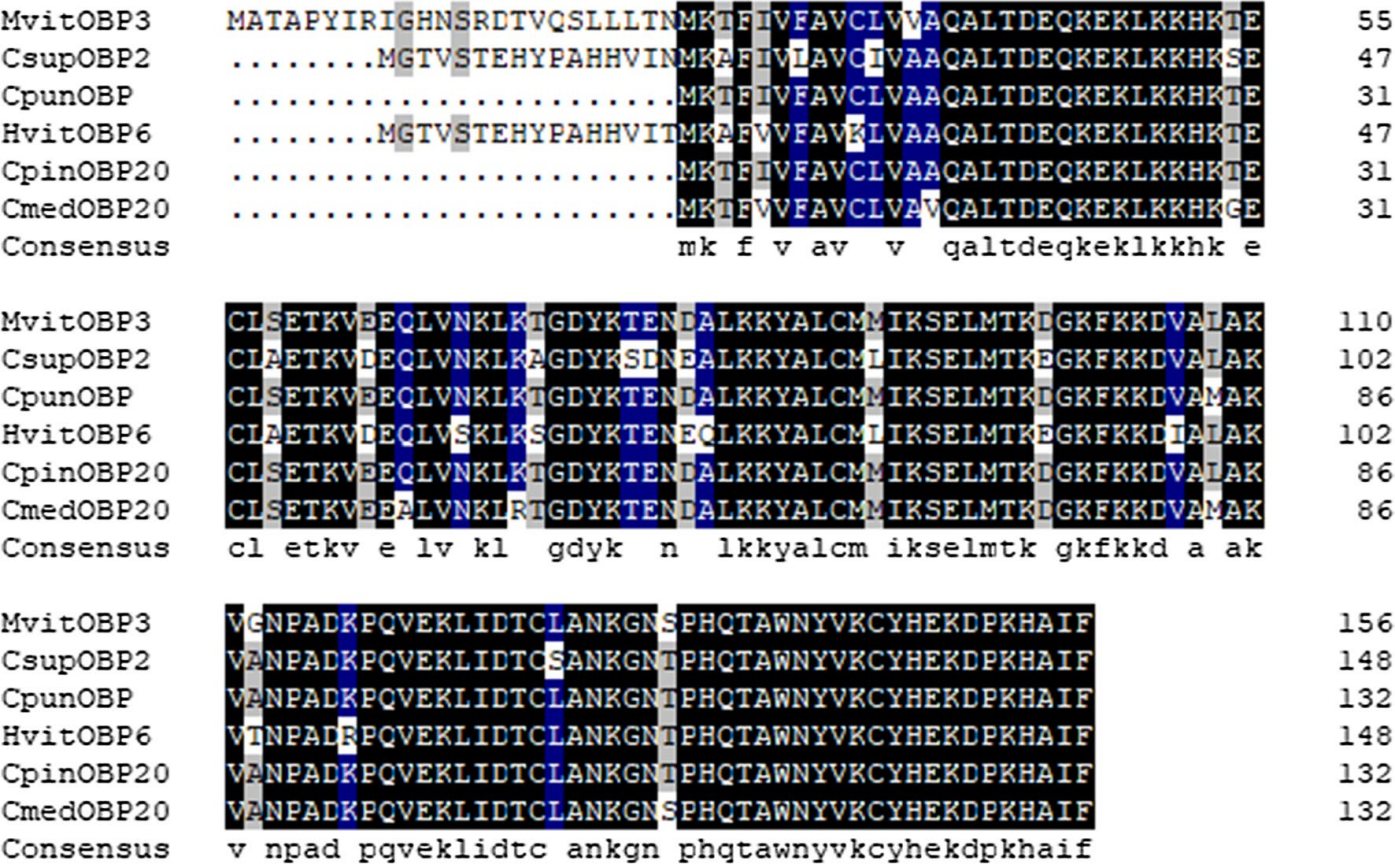
Multiple sequence alignment of OBPs from Lepidopteran insects including MvitOBP3.

The predicted molecular weight of MvitOBP3 protein was 17.79 kDa and included typical conserved six-cysteine signature, involving in the formation of three disulfide bridges and hydrophobic structure (Fig. 1). The ORF of *MvitOBP3* gene encoded 157 amino acids and the theoretical isoelectric point is 8.97. The phylogenetic tree analysis of OBP protein in Lepidoptera indicated that MvitOBP3, CsupOBP2 (RVE44133.1), CpunOBP (AMY16434.1), HvitOBP6 (AZB49387.1), CpinOBP20 (QEE82719.1) and CmedOBP20 (ALT31650.1) were clustered to one subgroup, highlighting their highly conserved characteristic in the CSP proteins family of Lepidoptera (Fig. 2). Multiple amino acid sequences alignment with other Lepidoptera OBPs also showed that MvitOBP3 had the highest sequence similarities with CsupOBP2 (RVE44133.1), CpunOBP, HvitOBP6, CpinOBP20 and CmedOBP20 (84.29%).

### Expression pattern of *MvitOBP3* gene

**Figure 3:**
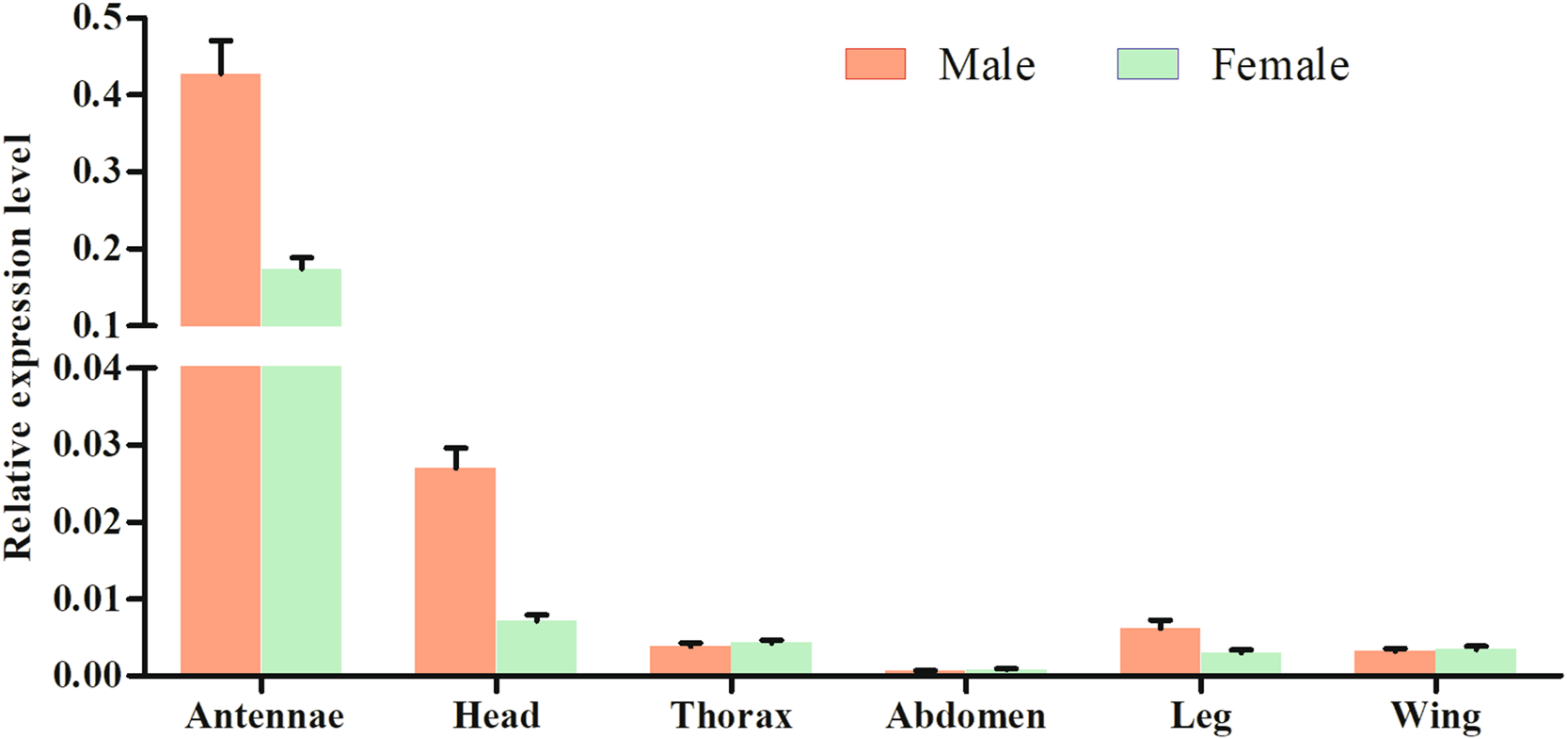
Relative transcript levels of *MvitOBP3* gene in different tissues of *M. vitrata*.

To understand the physiological function and tissue expression level of *MvitOBP2* gene, the transcript abundance of *MvitOBP3* in different tissues including antennae, head, thorax, abdomen, leg and wing were tested by using real-time PCR. The qPCR results indicated that expression level of *MvitOBP3* gene in antennae and head were significantly higher than that of other tissues (Fig. 3). Moreover, the transcript abundance of *OBP3* gene was the highest in male and female *M. vitrata* antennae, which was 15.8 times and 24.4 times higher than that of head tissues of male and female moths, respectively. In previous study, Real-time PCR analysis of *M. vitrata GOBP1-2* gene showed that their mRNA expressed level in the female antennae were higher than other tissues^7^. Based on the high expression pattern of *MvitOBP3* in antennae, we selected this gene for further investigation to test its plausible role in odor recognition.

### Purification of recombinant MvitOBP3 protein

**Figure 4:**
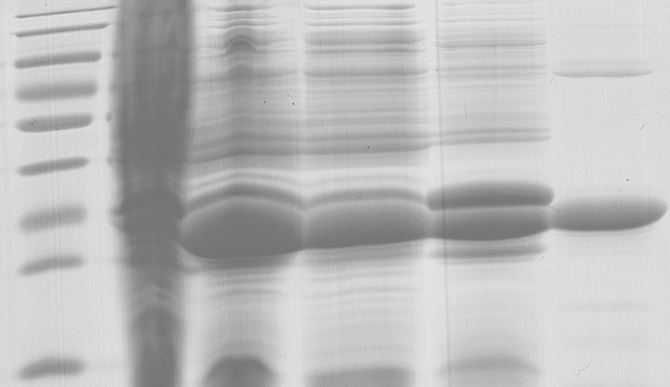
SDS-PAGE electrophoresis analysis of recombinant MvitOBP3. The lane is from left to right: marker protein, noninduced *E. coli*, induced *E. coli*, supernatant after broken, precipitation after broken, purified MvitOBP3.

As shown in Fig. 4, the recombinant MvitOBP3 protein was high expressed in the supernatant of *E. coli*, highlighting it could be abundantly expressed and purified in the prokaryotic expression system (Fig. 4). After ultrasonic crushing, the MvitOBP3 protein was purified by Ni ion affinity chromatography to obtain high pure protein and checked by sodium dodecyl sulfate–polyacrylamide gel electrophoresis (SDS-PAGE). Electrophoresis analysis demonstrated that there was an obvious target protein with high purity between the standard protein 25 KDa and 35 KDa (Fig. 4). The purified OBP3 protein was stored in − 80 °C for further test in the ligands binding experiments.

### Measurement of fluorescence binding affinities with host-plant volatiles

**Figure 5:**
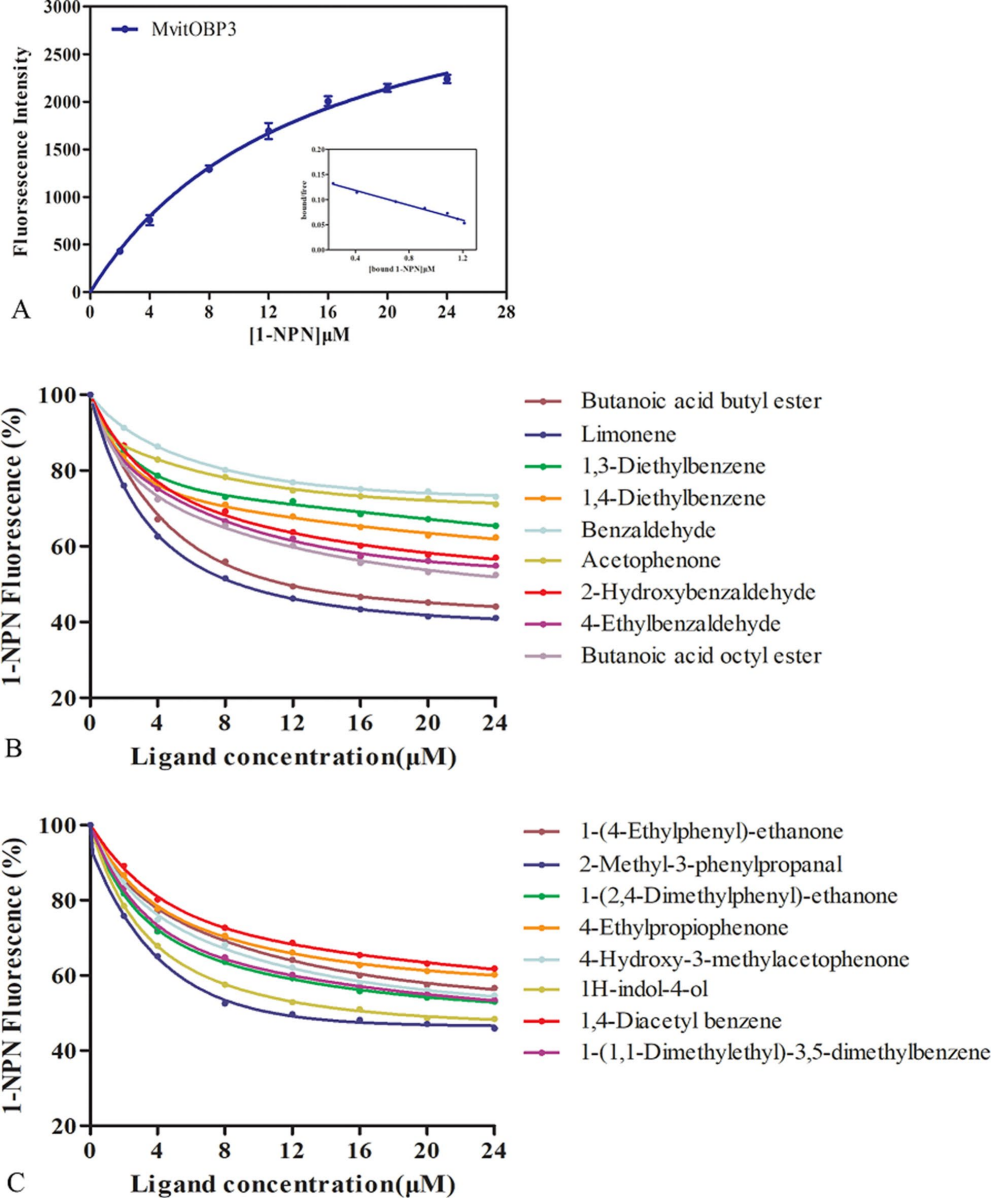
Competitive binding curves of host-plant volatile ligands to MvitOBP3 protein.

In fluorescence competitive binding assay, the 1-NPN was used as the fluorescent probe, and the dissociation constant (*K*_*i*_) values of the MvitOBP3/1-NPN complexes were calculated according to the changes in the fluorescence intensity (Fig. 5A). In previous study, there were seventeen volatile components were identified from host-plant *Vigna unguiculata* and *Lablab purpureus* and exhibited excellent antennal EAG and olfactory behavioral response^16^. These seventeen volatile ligands from host-plant were selected to test binding affinities of MvitOBP3 (Fig. 5 and Table 2). The fluorescence competitive binding results demonstrated that the MvitOBP3 protein had strong binding affinities to four volatile ligands, including butanoic acid butyl ester (9.79 µM), limonene (8.31 µM), 2-methyl-3-phenylpropanal (9.38 µM) and 1H-indol-4-ol (11.17 µM). The IC_50_ values of volatile ligands showed the concentration of ligand displacing 50% of the fluorescent probe, and IC_50_ values of butanoic acid butyl ester, limonene, 2-methyl-3-phenylpropanal and 1H-indol-4-ol with MvitOBP3 proteins were 11.07, 9.40, 10.61 and 12.64 μM, respectively (Table 2).

**Table 2:** The binding constants of different ligands. Binding of 1-NPN and different host-plant volatile ligands to MvitOBP3.

No	Compounds	MvitOBP3	
		IC_50_(μM)	*K*_i_(μM)
1	Butanoic acid butyl ester	11.07	9.79
2	Limonene	9.40	8.31
3	1,3-diethylbenzene	33.61	29.70
4	1,4-diethylbenzene	29.34	25.94
5	Benzaldehyde	43.3	38.30
6	Acetophenone	41.83	36.96
7	2-hydroxybenzaldehyde	24.19	21.38
8	4-ethylbenzaldehyde	22.76	20.13
9	Butanoic acid octyl ester	21.2	18.73
10	1-(4-ethylphenyl)-ethanone	24.07	21.27
11	2-methyl-3-phenylpropanal	10.61	9.38
12	1-(2,4-Dimethylphenyl)-ethanone	21.37	18.89
13	4-ethylpropiophenone	26.73	23.64
14	4-hydroxy-3-methylacetophenone	22.7	20.07
15	1H-indol-4-ol	12.64	11.17
16	1,4-diacetyl benzene	28.38	25.08
17	1-(1,1-Dimethylethyl)-3,5-dimethylbenzene	21.83	19.30

*Ki*, dissociation constant; IC_50_, ligand concentration displacing 50% of the fluorescence intensity of the MvitOBP /N-phenyl-1-naphthylamine complex.

Zhou et al. reported that the binding characteristics of *M. vitrata* GOBP1-2 proteins, which they mainly had great binding affinities with six volatile odorant molecules, such as butanoic acid butyl ester, limonene, 4-ethylpropiophenone, 1H-indol-4-ol, butanoic acid octyl ester and 2-methyl-3-phenylpropanal^7^. The ligands binding experiments indicated that MvitGOBP1 had better binding affinities with butanoic acid butyl ester, limonene, 4-ethylpropiophenone and 1H-indol-4-ol than other volatiles (*Ki* values: 12.52, 13.81, 14.19, 10.87 μM). And the MvitGOBP2 protein mainly recognize two volatile components, 2-methyl-3-phenylpropanal and butanoic acid octyl ester (*Ki* values: 3.81, 8.5 μM)7. These findings revealed that three OBP proteins could bind different essential flower volatile compounds from legume vegetables and involved in visiting flowers and spawning locations of *M. vitrata*. Besides, the PBP1-3 proteins were also identified from *M. vitrata* and they may play critical roles in the perception of sex pheromones of this legume pod borer^22^. Hence, these flower volatiles and four sex pheromones could be set as candidates for developing attractants to regulate behavior of plant seeking and host choice in *M. vitrata*. The present results might be used as an experimental basis for further integrated management strategies including efficient monitoring of field and interfering of host-locating behavior.

### Molecular docking

**Figure 6:**
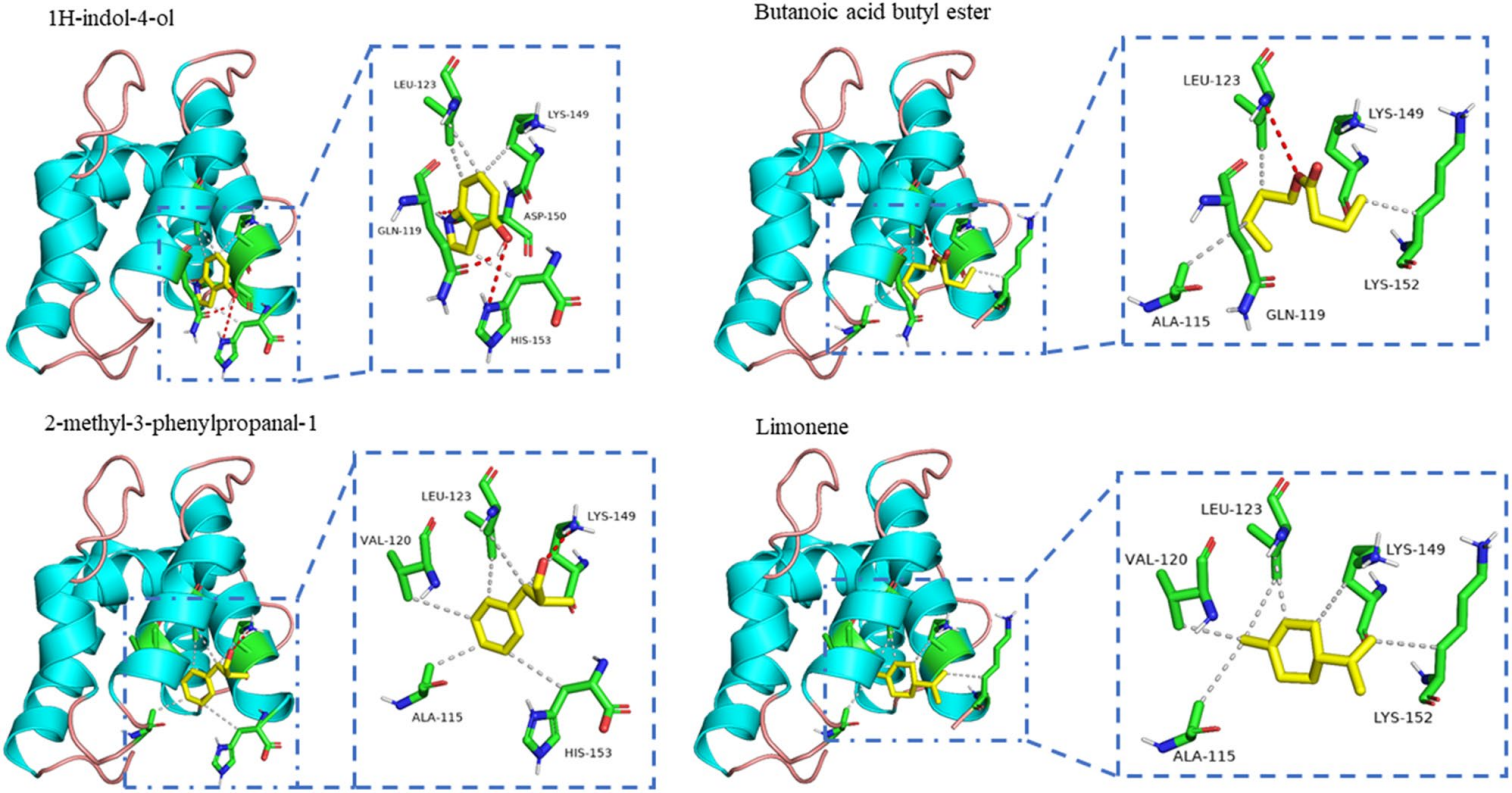
Molecular docking result of MvitOBP3 and host-plant volatile ligands.

As MvitOBP3 protein specifically binds to several host-plant volatile compounds from legumes, homology modeling and docking assays were performed to further explore the interactions of active odorants with MvitOBP3. The *P. regina* OBP56 protein was taken as the template for homology modeling. After the construction and optimization by Modeler 9.10 software, the final 3D model of MvitOBP3 was further checked. The dissociation constants for the top four best ligands for MvitOBP3, namely, Butanoic acid butyl ester, Limonene, 2-methyl-3-phenylpropanal and 1H-indol-4-ol were used for a docking assay with the 3D model of MvitOBP3 protein.Based on the molecular docking result (Fig. 6), hydrophobic interactions were the main linkage between MvitOBP3 and the four tested compounds. And the four ligand compounds showed above-threshold interaction with the tested MvitOBP3 protein. As shown in Table 3, there were many amino acids residues exhibited essential ligand-binding sites, such as Leu123 of Butanoic acid butyl ester, Lys149 of 2-methyl-3-phenylpropanal, and Gln119, Asp150 and His153 of 1H-indol-4-ol, which they involved in the hydrogen bonds formation between the MvitOBP3 protein with host-plant volatiles. After molecular docking analysis, site-directed mutagenesis and fluorescence competitive binding experiments will be used to verify docking results, which the key binding sites of OBP3 protein will be screened and obtained in next experiments.

**Table 3:** The Docking results of MvitOBP3 against different host-plant volatile ligands.

Compounds	CDOCKER Interaction energy (Kcal/mol)	Hydrophobic binding cavity site	Residues forming H-bond with ligand
Butanoic acid butyl ester	− 3.93	Ala115, Gln119, Leu123, Lys152	Leu123
Limonene	− 5.62	Ala115, Val120, Leu123, Lys149, Lys152	–
2-methyl-3-phenylpropanal	− 5.44	Ala115, Val120, Leu123, Lys149, His153	Lys149
1H-indol-4-ol	− 4.97	Leu123, Lys149, His153	Gln119, Asp150, His153

Besides, MvitOBP3 was primarily responsible for forming hydrophobic cavity including amino acid sites such as Ala115, Val120, Leu123, Lys149 and Lys152 to bind Limonene. Additionally, our computational analysis indicated lower binding energies between OBP3 with all the tested compounds (Table 3),which is consistent with IC_50_ values determined from the florescent binding experiments (Table 2). These results indicated that hydrogen bonds and hydrophobic cavity may play important roles in the olfactory recognition process of OBP3 protein to host-plant volatiles. A great number of studies reported that different subfamilies of insect OBPs were involved in recognition and transport of semiochemical components and olfactory signals through the hemolymph in chemosensory organs^9–14^. For instance, Li et al. found that Eastern honey bee, *Apis cerana* OBP2 had two key amino acids, Ser123 and Lys51 by docking analysis and site-directedmutagenesis, exhibiting important olfactory physiological function in visiting flower and pollination of Eastern honey bee^12^. Besides, another olfactory protein, chemosensory proteins also play an essential role in insect olfactory recognition, such as the oriental fruit moth, *Grapholita molesta* CSP8 protein could form hydrogen bond with 1-hexanol ligand through Thr27 and Leu30 amino acid sites using by site-directed mutagenesis and ligand-binding experiments^23^. In addition to fluorescence competitive binding assay, RNA interference and gene editing technique (Crispr/Cas9) have also been gradually applied to the molecular regulated research of insect olfaction in recent years^24,25^, which was beneficial to the application of integrated biological pest control in the field.

## Supplementary Information

Supplementary Information.
