# Supplementary material for: Functional characteristics of a novel odorant binding protein in the legume pod borer, Maruca vitrata
Source: Sci Rep. 2021 Jul 7;11:14027. doi: 10.1038/s41598-021-93382-7 (PMC8263619; doi:10.1038/s41598-021-93382-7)

Functional characteristics of a novel odorant binding protein in the legume pod borer, *Maruca vitrata*

Hui Ai [1], Yuying Liu [1], Guangyan Long [1], Yuan Yuan [1], Shaopei Huang [1], Yan Chen [2, *]

[1] Hubei Key Laboratory of Genetic Regulation and Integrative Biology, School of Life Sciences, Central China Normal University, Wuhan 430079, China.

[2] Wuhan Donghu University, Wuhan 430212, China.

The original image of OBP3 protein SDS-PAGE

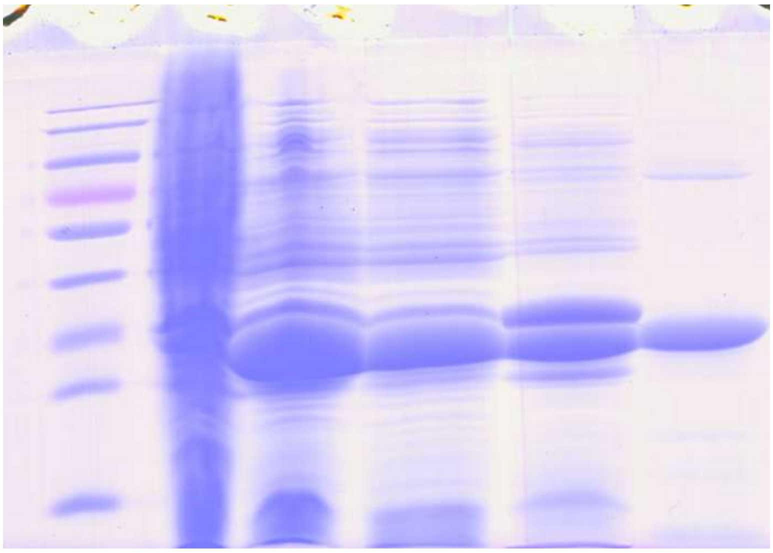

Supplement: Supplementary file 1 — Supplementary Information. [file 41598_2021_93382_MOESM1_ESM.pdf]
